# The Apoptotic Effect of D Rhamnose β-Hederin, a Novel Oleanane-Type Triterpenoid Saponin on Breast Cancer Cells

**DOI:** 10.1371/journal.pone.0090848

**Published:** 2014-03-06

**Authors:** Lin Cheng, Tian-Song Xia, Yi-Fen Wang, Wenbin Zhou, Xiu-Qing Liang, Jin-Qiu Xue, Liang Shi, Ying Wang, Qiang Ding

**Affiliations:** 1 Jiangsu Breast Disease Center, the First Affiliated Hospital with Nanjing Medical University, Nanjing, China; 2 State Key Laboratory of Phytochemistry and Plant Resources in West China, Kunming Institute of Botany, Chinese Academy of Sciences, Kunming, China; University of Quebec at Trois-Rivieres, Canada

## Abstract

There is growing interest in development of natural products as anti-cancer and chemopreventive agents. Many triterpenoids have been proved as potential agents for chemoprevention and therapy of breast cancer. Ginsenosides from *ginseng*, which mostly belong to dammarane-type triterpenoids, have gained great attention for their anti-breast cancer activity with diverse mechanisms. However, studies of other kinds of triterpenoid saponins on breast cancer are limited. Previously, we purified and identified a novel oleanane-type triterpene saponin named D Rhamnose β-hederin (DRβ-H) from *Clematis ganpiniana,* a Chinese traditional anti-tumor herb. In the present study, DRβ-H showed strong inhibitory activity on the growth of various breast cancer cells and induced apoptosis in these cells. DRβ-H inhibited PI3K/AKT and activated ERK signaling pathway. PI3K inhibitor LY294002 synergistically enhanced DRβ-H-induced apoptosis whereas MEK inhibitor U0126 reduced the apoptosis rate. Moreover, DRβ-H regulated the ratio of pro-apoptotic and anti-apoptotic Bcl-2 family proteins. Furthermore, DRβ-H induced depolarization of mitochondrial membrane potential which released Apaf-1 and Cytochrome *C* from the inter membrane space into the cytosol, where they promoted caspase-9 and caspase-3 activation. This is the first report on the pro-apoptotic effects of DRβ-H, a novel oleanane-type triterpenoid saponin, on breast cancer cells and its comprehensive apoptosis pathways. It implied that oleanane-type triterpenoid saponin DRβ-H could be a promising candidate for chemotherapy of breast cancer.

## Introduction

Breast cancer is a worldwide malignant disease and a major cause of death in women [1]. Many anti-cancer agents have shown efficacy in extending the survival of breast cancer patients. Although disease-free survival and overall survival have been significantly improved, there are still some patients died of breast cancer after systemic therapies, especially for advanced breast cancer [2]. Using compounds from natural plants as potential cancer preventive and/or therapeutic agents has become a fascinating strategy [3], [4]. Identification and investigation of active components from natural plants are important for assessing their potential for clinical use. A large number of components purified from herbs have been used to curing various cancers including breast cancer. For example, paclitaxel (TAXOL), a natural chemotherapeutic drug isolated from the bark of the *pacific yew*, is currently used widely for treating breast cancer [5]. Therefore, development of new therapeutic agents from natural source has great promise for breast cancer treatment.

We have purified and identified four kinds of triterpenoids derivatives from *Clematis ganpiniana.* They showed cytotoxicity against breast cancer cells [6]. One of them was D Rhamnose β-hederin (3β-[(α-L-arabinopyranosyl)-oxy] olean-12-en-28-oicacid) (DRβ-H), which belonged to triterpenoid saponins. Triterpenoid saponins are an important class of natural products and distributed widely in plant kingdom [7], [8]. Several excellent studies provided an overview of the triterpenoids as potential agents for chemoprevention and therapy of breast cancer [9], [10]. Triterpenoid saponins are further classified into two major sub-classes: tetracyclic and pentacyclic triterpenoid saponins according to the skeletal structures of the aglycones [11], [12]. Previous studies found that triterpenoid saponins showed inhibitory effect on various cancer cells by regulating different pathways, for example, EGFR [13], ER [14], Fas/FasL [15], PI3K/AKT [14] and MAPK pathways [16]. Ginsenoides from *ginseng*, most of which are dammarane-type triterpenoid saponins [17], belong to tetracyclic triterpenoids. Ginsenosides are one of the most widely recognized triterpenoid saponins and have attracted great attention worldwidely on the anti-breast cancer activity with diverse underlying mechanisms [18]–[21]. However, studies on other kinds of triterpenoid saponins such as oleanane-type triterpenoids are scarce till now, most of which focus on biological activity [22], [23], while the mechanisms have not been widely reported yet. DRβ-H was one of oleanane-type triterpenoid derivatives and belonged to pentacyclic triterpenoid saponins. Then structures of DRβ-H and ginsenoides have different skeleton and may have definitely different bioactivities.

DRβ-H have been previously reported to inhibit growth and induce apoptosis of breast cancer cells [6], however, further effects and mechanisms of DRβ-H on breast cancer is currently unavailable. In this study, we evaluated effects of DRβ-H on growth and apoptosis of various human breast cancer cell lines, and explored the underlying molecular mechanisms.

## Materials and Methods

### Drug Preparations

Protocols of the collection, storage, extraction of the plant material of *Clematis ganpiniana*, the methods of the purification and analysis of the DRβ-H were described in the previous study [6].

### Cell Culture

The human breast cancer cell lines MCF-7, MDA-MB-231, BT474, SUM1315 and normal mammary epithelial cell line MCF-10A were obtained from American Type Culture Collection (Manassas, USA) and incubated in a humidified atmosphere of 5% CO_2_ in air at 37°C and fed with the culture medium of High glucose Dulbecco’s Modified Eagle Medium, supplemented with 10% fetal bovine serum, 1% penicillin–streptomycin solution. For routine passages, cultures were split 1∶3 when they reached 80–90% confluence generally every 2–3 days. All experiments were performed on exponentially growing cells. Four breast cancer cell lines MCF-7, MDA-MB-231, BT474 and SUM1315, which represented different phenotypes of this heterogeneous disease, were used to evaluate the growth inhibition of DRβ-H. MCF-7 and MDA-MB-231 cells were widely used in studies on human breast cancer. In this study, we used these two cell lines to explore the underlying molecular mechanisms of DRβ-H.

### MTT Assay

The MTT assay was used to measure the inhibition growth of DRβ-H in breast cancer cell lines and MCF-10A cells. Briefly, 5×10^3^ cells were seeded into a 96-well plate in triplicate and 8 h later DRβ-H was added into the wells at the indicated final concentrations (5, 10, 20, 40 and 80 µg/ml), while cells cultured in medium with 0.05% DMSO as a negative control. After incubation with DRβ-H for 24 h, 48 h and 72 h, the medium in each well was replaced with 20 µl of MTT at 5 mg/ml final concentration, and 4 h later 150 µl DMSO/well was added to dissolve the formed violet formazan crystals within metabolically viable cells. The plates were incubated at room temperature for 15 min and then read at 490 nm with a microplate reader (5082 Grodig, TECAN, Austria). Percent of growth inhibition was calculated as (OD of the control - OD of the experiment samples)/OD of the control × 100.

### Colony Formation Assay

The effect of DRβ-H on breast cancer colony formation was tested. Breast cancer cells were plated in six-well plates at a density of 500 cells/well. After 8 h, the cells were treated with different concentrations (20, 30 and 40 µg/ml) of DRβ-H for two weeks. Then medium was removed and cells were washed twice with PBS and stained for 15 minutes using Giemsa solution, rinsed with tap water, and dried at room temperature. The colonies in each well were counted, and all cell colonies contained 50 or more cells.

### Apoptosis Analysis by Flow Cytometry

After exposure to DRβ-H at concentrations indicated (20, 30 and 40 µg/ml) for 48 h, breast cancer cells and MCF-10A cells were washed twice with PBS at 4°C, resuspended in stain containing AnnexinV-FITC and propidium iodide (PI) (Carlsbad, CA, USA) for 15 min incubation on ice, and analyzed with FACSAria flow cytometer (Becton Dickinson, San Jose, CA, USA) using FACSDiva software. For the PI3K and MEK inhibition study, the cells were pre-incubated with PI3K inhibitor LY294002 (10 µM) or MEK inhibitor U0126 (20 µM) 1 h before DRβ-H treatment. Approximately 10^5^ cells were analyzed for each treatment.

### Measurement of the Mitochondrial Membrane Potential (ΔΨm) with JC-1

The mitochondrial membrane potential was measured according to the manufacturer’s instruction with JC-1 (Carlsbad, CA, USA). After exposure to DRβ-H for 2 h, 6 h and 12 h, cells were washed twice with PBS, incubated in the working solution of 2 µg/ml JC-1 for 30 min at 37°C in 5% CO_2_ atmosphere, and observed with EISS LSM 5 LIVE confocal microscope (Jena, Germany). The fluorescence was measured at an excitation: emission of 485/538 for green monomers and at an excitation: emission of 485/590 for red aggregates. Valinomycin was used at a concentration of 0.1 µM as a positive control for depolarization of the ΔΨm.

### Measurement of Cellular Caspases-3, -8 and -9 Activities

Caspases-3, -8 and -9 activities were quantified by measuring cleavage of the colorimetric peptides RED-DEVD-FMK, RED-IETD-FMK and RED-LEHD-FMK, respectively (Biovision, Palo Alto, CA, USA). Briefly, at the end of designated treatment (48 h of exposure to 30 µg/ml DRβ-H ), equal numbers of control or treated cells were incubated with RED-DEVD-FMK, RED-IETD-FMK and RED-LEHD- FMK respectively (2 µg/ml) for 20 min at 37°C in 5% CO_2_ atmosphere, then washed twice by PBS and analyzed with FACSAria flow cytometer. For the caspase inhibition study, the cells were pre-incubated with inhibitors: z-DEVD-FMK, z-IETD-FMK, and z-LEHD-FMK (respectively caspase-3, -8 and -9 inhibitors) 1 h before DRβ-H treatment.

### Western Blotting Analysis

MCF-7 and MDA-MB-231 cells were seeded at 1×10^6^ cells in 100 mm^2^ dishes. Cells were treated in complete medium with DRβ-H at the times indicated. For the PI3K and MEK inhibition study, the cells were pre-incubated with PI3K inhibitor LY294002 (10 µM) or MEK inhibitor U0126 (20 µM) 1 h before DRβ-H treatment. After treatment, adherent cells were gently scraped from the plates into the medium containing floating cells to obtain all cells. Cells were then centrifuged, washed in PBS, lysed in ice-cold lysis buffer containing phosphatase inhibitor cocktail and protease inhibitor cocktail (Boehringer Mannheim, Germany) to obtain total protein. Protein concentrations were determined using the Bradford method.

Apaf-1 and Cytochrome *C* in mitochondrial fraction were analyzed by isolation of mitochondrial protein using the Cell Mitochondria Isolation Kit (Beyotime, Biotech, Peking, China). Briefly, after exposure, MCF-7 and MDA-MB-231 cells were harvested and centrifuged at 800 g at 4°C for 10 min. The pellets were added with 20 mM N-2-hydroxyethylpiperazine-N0-20-ethanesulfonic acid (HEPES) buffer containing protease inhibitor cocktail and disrupted with a glass tissue grinder. Homogenates were centrifuged at 800 g at 4°C for 10 min, and the resulting supernatants were transferred to 0.5 ml conical tubes, and further centrifuged at 10 000 g at 4°C for 20 min. The final pellets, containing the mitochondrial fraction, were analyzed for protein content using the Bradford method. Cell lysates were electrophoresed through 10–12% SDS-PAGE gel, and transferred to PVDF membranes, which were activated in methanol. The blots were probed or reprobed with antibodies. GAPDH was used to normalize for protein loading. The membranes were probed using ECL and autoradiographed. The intensity of the bands was determined using densitometric analysis. The primary antibodies used were anti-rabbit PI3K, p-PI3K (Tyr458), PDK1, p-PDK1 (Ser241), AKT, p-AKT (Ser473), ERK1/2, p-ERK1/2 (Thr202/Tyr204), JNK, p-JNK (Thr183/Tyr185), P38, p-P38 (Thr180/Tyr182). PI3K inhibitor LY294002, MEK1/2 inhibitor U0126, Bcl-2 family Kit and the primary antibodies described above were purchased from Cell Signaling technology. Purified Mouse Anti-Human apoptotic protease activating facter-1 (Apaf-1) and Cytochrome *C* were purchased from BD Bioscience. GAPDH was from Sigma. Anti-rabbit and Anti-mouse secondary antibodies were from Cell Signaling technology. The antibodies were diluted according to the manufacturer’s instructions. A table including blocking steps, wash steps, dilution, and incubation duration and temperature for primary and secondary antibodies was provided (Table S1).

### Statistical Analysis

The data were analyzed using the SPSS 20.0 software. For all the measurements, oneway ANOVA followed by Bonferroni test was used to assess the statistically significance of difference between control and groups-treated. A statistically significant difference was considered at the level of *p*<0.05.

## Results

### DRβ-H Inhibited the Growth of Breast Cancer Cells

**Figure 1:**
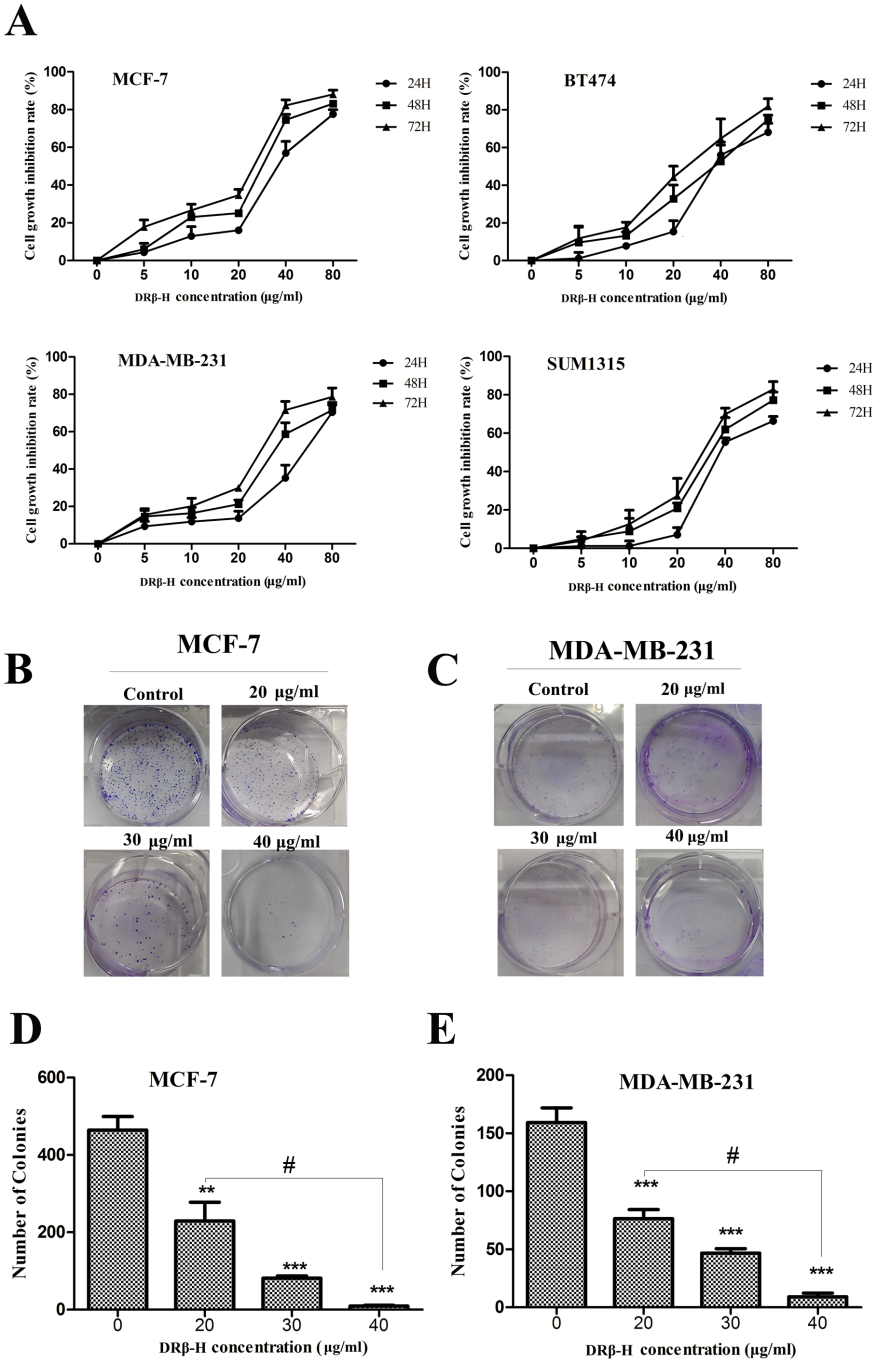
DRβ-H inhibited growth of various breast cancer cells. (A) MTT assay of MCF-7, MDA-MB-231, BT474 and SUM1315 cells treated by DRβ-H. (B, C) Colony formation assay of MCF-7 and MDA-MB-231 cells following treatment with DRβ-H for two weeks. (D, E) Colony numbers of three independent experiments were shown in column statistics. Data are mean±standard error of mean (SEM) of three independent experiments. *p<0.05, **p<0.01, ***p<0.001 vs. DRβ-H -untreated group. The # shows that the two groups had statistically significant difference.

In this study, four breast cancer cell lines MCF-7, MDA-MB-231, BT474, SUM1315 and nomal cell line MCF-10A were used. The inhibitory rate of growth was determined by MTT assay. DRβ-H showed inhibition in all the four breast cancer cell lines in both dose-dependent and time-dependent manners which were statistically significant compared to the negative control (*p*<0.05) (Figure 1A). DRβ-H showed inhibition in MCF-10A cells (*p*<0.05) (Figure S1A). However, the effect was weaker than all of the four cancer cell lines (Figure S1B).

Similarly, the colony formation assay showed dose-dependent inhibition of colony formation by DRβ-H, further confirming the cell growth inhibition effect of DRβ-H (Figure 1B, C, D, E).

### DRβ-H Induced Apoptosis in Breast Cancer Cells

**Figure 2:**
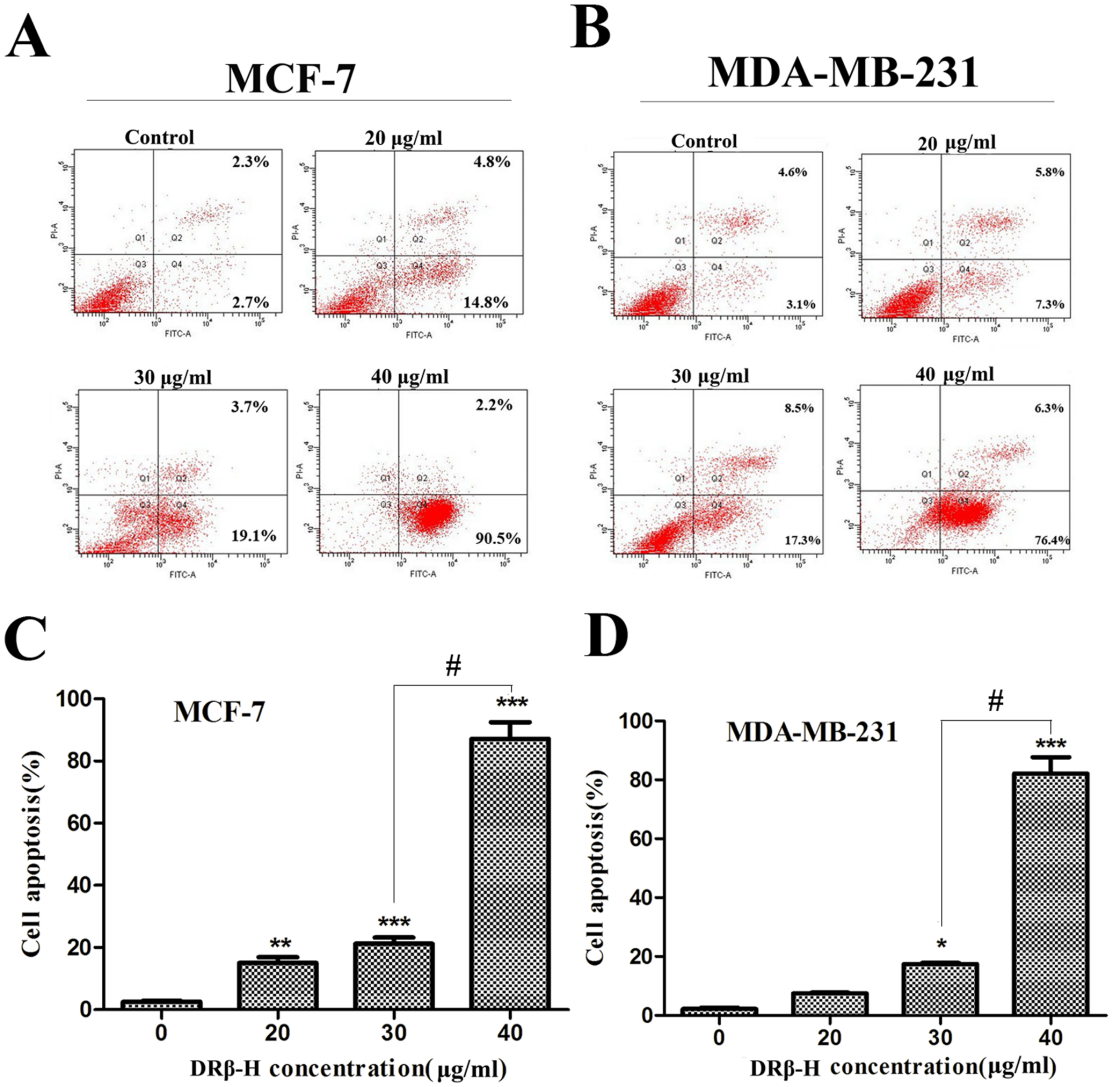
DRβ-H induced apoptosis in breast cancer cells. (A, B) The apoptosis rate of MCF-7 and MDA-MB-231 cells measured by flow cytometry. DRβ-H induced early apoptosis in MCF-7 and MDA-MB-231 cells in a dose-dependent manner. (C, D) Early apoptosis cells of three independent experiments were shown in column statistics. Data are mean±SEM of three independent experiments. *p<0.05, **p<0.01, ***p<0.001 vs. DRβ-H-untreated group. The # shows that the two groups had statistically significant difference.

The apoptosis rate was measured by flow cytometry. MCF-7 and MDA-MB-231 treated with DRβ-H for 48 h were first double-stained with Annexin V and PI, and then analyzed by flow cytometry. In cells treated with DRβ-H, we detected a major increase in the Annexin V^+^/PI^−^ fraction (regarded as early apoptotic) subpopulations, whereas Annexin V^+^/PI^+^ fraction (regarded as necrotic) subpopulations had little change. The activity was dose-dependent and more effective in MCF-7. After incubated with 40 µg/ml DRβ-H for 48 h, early apoptosis rate of MCF-7 and MDA-MB-231 cells were significantly increased up to 90.5% and 76.4%, respectively (Figure 2A, B, C, D). DRβ-H induced apoptosis of MCF-10A cells (Figure S1C, D). However, the effect was weaker than all of the four cancer cell lines. (Figure S1E).

### DRβ-H Affected the Mitochondrial Membrane Potential (ΔΨm) of Breast Cancer Cells

**Figure 3:**
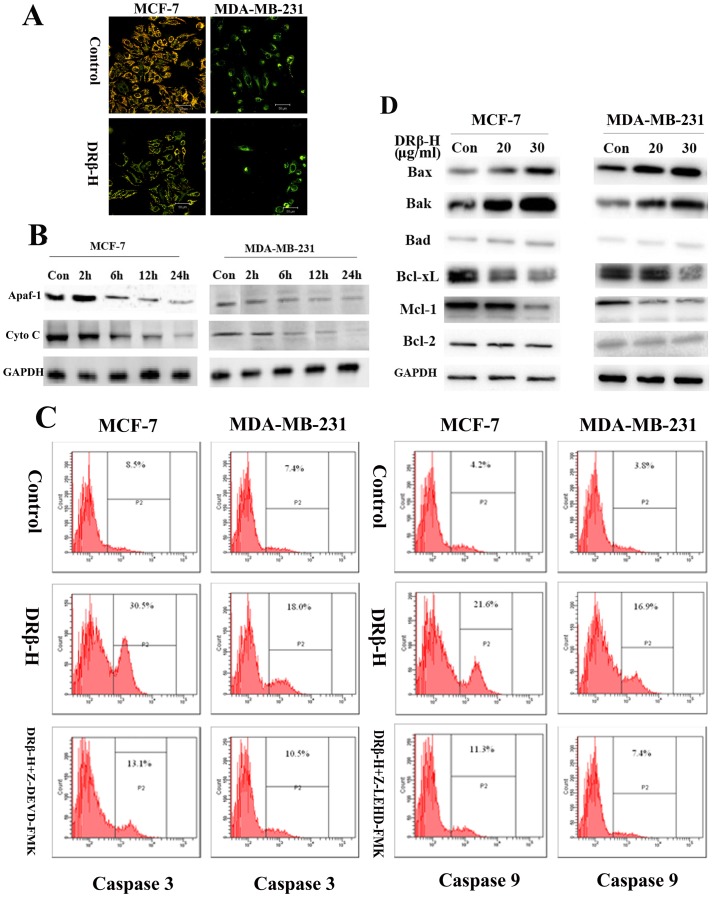
DRβ-H induced mitochondria-mediated apoptosis of MCF-7 and MDA-MB-231 cells. (A) Effects of DRβ-H on the mitochondrial depolarization in MCF-7 and MDA-MB-231 cells. After the application of DRβ-H, JC-1 fluorescence shifted from red-orange to greenish yellow, which indicated the depolarization of mitochondrial membrane potential. (B) Effect of DRβ-H on Apaf-1 and Cytochrome *C* release. Cyto C: Cytochorme *C*. (C) Effect of DRβ-H on caspase-3 and caspase-9 activation. DRβ-H increased the activity of caspase-3 and caspase-9 in both MCF-7 and MDA-MB-231 cells. This activation could be reversed by the caspase inhibitors respectively. (D) Effect of DRβ-H on the expression of Bax, Bak, Bad, Bcl-xL, Mcl-1 and Bcl-2. Data are mean±SEM of three independent experiments. *p<0.05, **p<0.01, ***p<0.001 vs. DRβ-H-untreated group.

MCF-7 and MDA-MB-231 cells were treated for 6 h with DRβ-H (30 µg/ml), and then mitochondrial membrane potential was measured. After the application of DRβ-H, JC-1 fluorescence shifted from red-orange to greenish yellow, which indicated the depolarization of mitochondrial membrane potential (Figure 3A). Mitochondrial membrane potential ΔΨm of cells treated with DRβ-H of three independent experiments were shown in column statistics (Figure S2A).

### DRβ-H Regulated the Apaf-1 and Cytochrome *C* Release

MCF-7 and MDA-MB-231 cells were treated for 2 h, 6 h, 12 h or 24 h with DRβ-H (30 µg/ml) and both mitochondrial Apaf-1 and Cytochrome *C* level were detected by Western blotting. DRβ-H decreased both mitochondrial Apaf-1 and Cytochrome *C* expressions in a time-dependent manner (Figure 3B). Expressions of mitochondrial Apaf-1 and Cytochrome *C* of cells treated with DRβ-H of three independent experiments were shown in column statistics (Figure S2B, C).

### DRβ-H Regulated Caspase-3 and Caspase-9 Activation

After exposure to DRβ-H (30 µg/ml) for 48 h, activity of caspase-3 and caspase-9 was increased in both MCF-7 and MDA-MB-231 cells. This activation could be reversed by the caspase inhibitors (Figure 3C). Caspase-3, caspase-9 positive rate of cells treated with DRβ-H with/without caspase inhibitors of three independent experiments were shown in column statistics (Figure S2D, E). However, DRβ-H did not affect caspase-8 in breast cancer cells (Data not shown).

### DRβ-H Regulated Bcl-2 Family Proteins

We measured the expression of Bcl-2 family proteins by Western blotting. After exposure to DRβ-H (20 µg/ml and 30 µg/ml) for 48 h, DRβ-H induced expression of pro-apoptotic proteins Bax and Bak whereas inhibited expression of anti-apoptotic proteins Bcl-xL, Mcl-1 in both the cell lines in a dose-dependent manner. However, the expression of pro-apoptotic proteins Bad and anti-apoptotic proteins Bcl-2 showed little change (Figure 3D). So the ratio of Bax/Bcl-2 expression was significantly increased after DRβ-H treatment in MCF-7 and MDA-MB-231 cells. Expressions of Bcl-2 family proteins of cells treated with DRβ-H of three independent experiments were shown in column statistics (Figure S2F, G).

### DRβ-H Regulated the PI3K/AKT Pathway in Breast Cancer Cells

**Figure 4:**
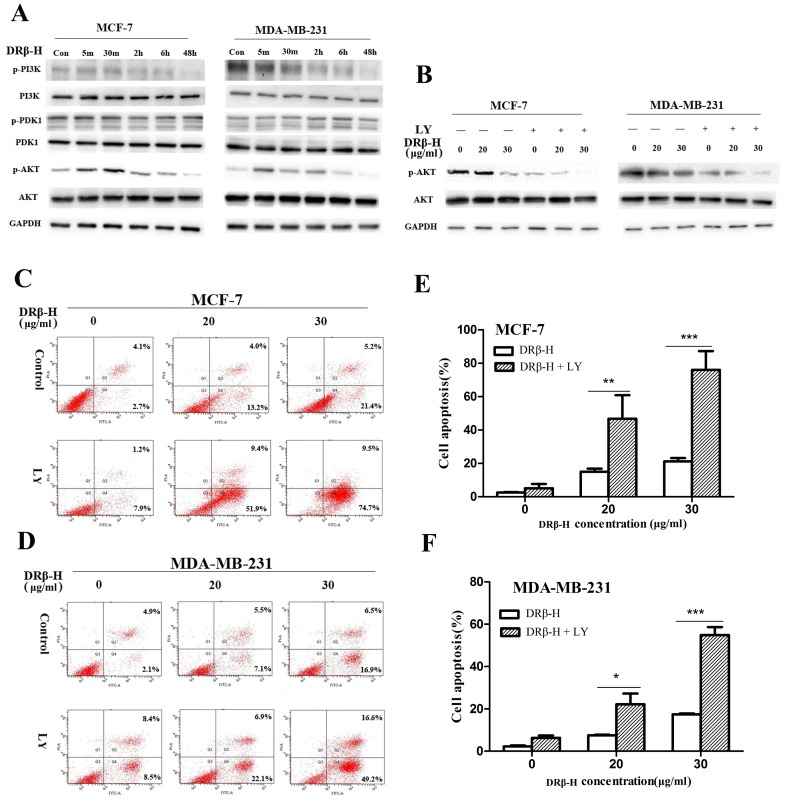
DRβ-H induced apoptosis of MCF-7 and MDA-MB-231 cells by inhibiting PI3K/AKT signaling pathway. (A) Effect of DRβ-H on the phosphorylation of PI3K, PDK1, and AKT in MCF-7 and MDA-MB-231 cells. (B) LY294002 enhanced the DRβ-H-induced inhibition of p-AKT. (C, D) LY294002 markedly increased the DRβ-H-induced apoptosis rate in MCF-7 and MDA-MB-231 cells. (E, F) Early apoptosis rate of cells treated with DRβ-H and/or LY294002 of three independent experiments were shown in column statistics. LY: LY294002. Data are mean±SEM of three independent experiments. *p<0.05, **p<0.01, ***p<0.001 vs. DRβ-H-treated alone group.

Western blotting revealed the decrease in the expression of the p-PI3K after 2 h of 30 µg/ml DRβ-H treatment in MCF-7 and MDA-MB-231 cells in a time-dependent manner, and this suppression was sustained for 48 h, whereas a slight increase of p-AKT was found at the early time points (5 min-2 h). However, DRβ-H markedly decreased phosphorylated AKT at late time point (48 h), hence we focused on the PI3K/AKT signaling to account for its inhibitory mechanism. Moreover, the expression of p-PDK1, PDK1, PI3K, AKT showed little change (Figure 4A). Expressions of PI3K, p-PI3K, PDK1, p-PDK1, AKT, and p-AKT of cells treated with DRβ-H of three independent experiments were shown in column statistics (Figure S3A, B).

### PI3K Inhibitor LY294002 Synergistically Enhanced DRβ-H-induced Apoptosis through Inhibition of AKT Activation

Since the activation of AKT is regulated by PI3K, we examined the influence of LY294002 in phosphorylation of AKT. MCF-7 and MDA-MB-231 cells were pretreated by 10 µM LY294002 for 1 h before DRβ-H treatment. The phosphorylation of AKT at Ser473 was inhibited by the treatment of PI3K inhibitor LY294002. Moreover, p-AKT in these cells was dramatically reduced under the pretreatment of LY294002 compared to treatment by DRβ-H alone (Figure 4B). Expressions of AKT, and p-AKT of cells treated with DRβ-H and/or LY294002 for 48 h of three independent experiments were shown in column statistics (Figure S3C, D).

Such cells were also subjected to apoptosis assay. LY294002 pretreatment further enhanced DRβ-H-induced apoptosis in MCF-7 and MDA-MB-231 cells (Figure 4C, D). Apoptosis rate of cells treated with DRβ-H and/or LY294002 for 48 h of three independent experiments were shown in column statistics (Figure 4E, F).

### DRβ-H Regulated the p-ERK Expression in Breast Cancer Cells

**Figure 5:**
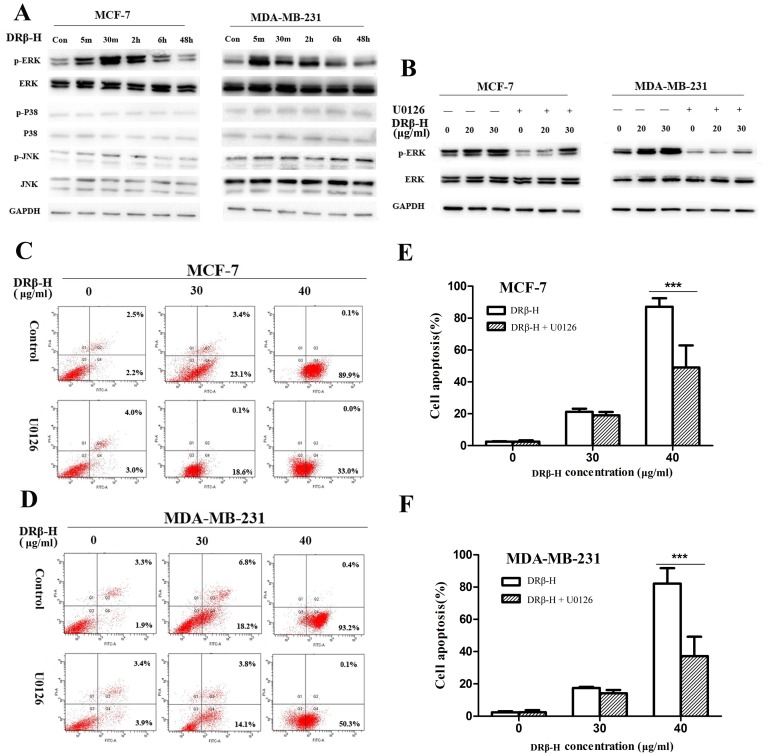
DRβ-H induced apoptosis of MCF-7 and MDA-MB-231 cells by activating ERK. (A) Effect of DRβ-H on the phosphorylation of ERK, P38 and JNK in MCF-7 and MDA-MB-231 cells. (B) U0126 reduced the DRβ-H-induced activation of p-ERK. (C, D) U0126 significantly reduced the DRβ-H–induced apoptosis rate in MCF-7 and MDA-MB-231 cells. (E, F) Early apoptosis rate of cells treated with DRβ-H and/or U0126 of three independent experiments were shown in column statistics. Data are mean±SEM of three independent experiments. *p<0.05, **p<0.01, ***p<0.001 vs. DRβ-H-treated alone group.

Western blotting was performed to determine whether the MAPK cascades including JNK, ERK1/2 and P38 were involved in DRβ-H induced apoptosis. DRβ-H up-regulated the phosphorylation of ERK1/2 in MCF-7 and MDA-MB-231 cells from 5 min to 6 h after treatment of 30 µg/ml DRβ-H and then returned to normal, while p-JNK and p-P38 showed no significant change under DRβ-H application (Figure 5A). Expressions of ERK, p-ERK, JNK, p-JNK, P38, p-P38 of cells treated with DRβ-H of three independent experiments were shown in column statistics (Figure S4A, B).

### MEK Inhibitor U0126 Reduced DRβ-H-induced Apoptosis through Inhibition of ERK Activation

To further verify whether ERK1/2 phosphorylation was necessary for DRβ-H-induced apoptosis, we used MEK inhibitor U0126 to assess the activation of these kinases. Not surprisingly, the phosphorylation of ERK was inhibited by the treatment of MEK inhibitor U0126. As expected, DRβ-H stimulated a phosphorylation of ERK1/2 in MCF-7 and MDA-MB-231 cells at 30 min whereas p-ERK in these cells was dramatically reduced under the pretreatment of U0126 compared to treatment by DRβ-H alone (Figure 5B). Expressions of p-ERK of cells treated with DRβ-H and/or U0126 for 30 min of three independent experiments were shown in column statistics (Figure S4C, D).

Cells under pretreatment with U0126 for 1 h and then stimulated with 30 and 40 µg/ml DRβ-H for 48 h were also subjected to apoptosis assay. U0126 pretreatment reduced DRβ-H-induced apoptosis in MCF-7 and MDA-MB-231 cells (Figure 5C, D). Apoptosis rate of cells treated with DRβ-H and/or U0126 for 48 h of three independent experiments were shown in column statistics (Figure 5E, F). The apoptosis rate of cells treated by 20 µg/ml DRβ-H and U0126 showed no significant different to cells treated with DRβ-H alone (Data not shown).

## Discussion

We reported that DRβ-H, a novel oleanane-type triterpenoid saponin, had strong inhibitory activity on different breast cancer cells. DRβ-H was found to induce apoptosis in both the ER^+^ human breast cancer cell line MCF7 and ER^−^ breast cancer cell line MDA-MB-231. However, the effect of DRβ-H on normal mammary epithelial cell, MCF-10A was weaker than breast cancer cell lines. DRβ-H induced mitochondria-mediated apoptosis of MCF-7 and MDA-MB-231 and regulated expression of Bcl-2 family proteins. Moreover, DRβ-H induced apoptosis by inhibiting PI3K/AKT pathway and activating ERK pathway.

In DRβ-H-induced apoptosis, caspase-3 and caspase-9 were involved, while no obvious activation of caspase-8 was detected. JC-1 staining was taken to detect the membrane potential of mitochondria. The membrane potential of mitochondria in breast cancer cells was greatly reduced by DRβ-H, and Apaf-1 and Cytochrome *C* were released from the mitochondria to cytoplasm. All these results indicated that DRβ-H induced apoptosis in breast cancer cells through the mitochondrial pathway. Mitochondria play a central role in apoptotic process. Both extrinsic pathway and intrinsic pathway can converge at the mitochondrial level and trigger mitochondrial membrane permeabilization [24]. Mitochondrial apoptotic pathway was reported widely for the actions of triterpenoid saponins in other human cancers including liver cancer [25]–[27], gastric cancer [28], esophageal cancer [29], colorectal cancer [30] and so on. However, reports about mitochondrial apoptotic activity in breast cancer of triterpenoid saponins are scarce, most of which focus on ginsenosides. For example, Ginsenoside Rh2 inhibited viability of both MCF-7 and MDA-MB-231 human breast cells by mitochondrial pathway [19]. Ginsenoside F2 induced apoptosis in breast cancer stem cells via mitochondria pathway [20]. Moreover, α-Hederin, a pentacyclic triterpenoid saponin similar to DRβ-H, induced apoptosis via mitochondrial perturbations [31], substantiating our findings as a possible common mechanistic pathway of triterpenoid saponins-induced apoptosis.

Any imbalance of expression levels of anti- and pro-apoptotic Bcl-2 family proteins will disrupt the integrity of the outer mitochondrial membrane [32], [33]. DRβ-H treatment induced remarkable up-regulation of Bax and Bak expression, while Bcl-2 protein levels showed no significant changes, and decreased of Bcl-xL and Mcl-1, suggesting that changes in the ratio of pro-apoptotic and anti-apoptotic Bcl-2 family proteins might contribute to the apoptosis promotion activity of DRβ-H. The apoptotic effect is more dependent on the balance of Bax and Bcl-2 than on Bcl-2 quantity alone [34]. Typically, the ratio of Bax and Bcl-2 protein expression is used as an index of apoptosis [35]. The regulatory effects of DRβ-H on the Bcl-2 family are correlated with the release of Apaf-1 and Cytochrome *C* from the mitochondria into the cytoplasm and the activation of caspase 3 and caspase 9. Previous studies also demonstrated that regulation of Bcl-2 family molecules and mitochondria apoptotic pathway involved in triterpenoid saponins-induced apoptosis in human breast cancer cells [16], [19], liver cancer cells [25], [26], colon cancer cells [36] and so on. Like most kinds of triterpenoid saponins, expecially ginsenosides, DRβ-H exerted apoptotic effect on breast cancer cells through mitochondrial apoptotic pathway as well as regulation of Bcl-2 family.

DRβ-H treatment inhibited the expression of p-PI3K after 2 h. A slight increase of p-AKT was found at the early time points and the compound markedly decreased p-AKT at late time point, whereas no significant change was found in the expression of p-PDK1. So DRβ-H might inhibit PI3K/AKT pathway after a relatively long time without passing from PDK1. Furthermore, PI3K inhibitor LY294002 synergistically enhanced DRβ-H-induced apoptosis through inhibition of AKT activation. Our findings suggested DRβ-H induced apoptosis of breast cancer cells by inhibiting PI3K/AKT signaling pathway. PI3K/AKT pathway is an attractive target for anticancer agents [37]. Reports about PI3K/AKT activity in breast cancer of triterpenoid saponins are limited, most of which focusing on ginsenosides. For example, Ginsenoside Rp1 could exhibit anti-cancer activity by reducing p-AKT expression of MCF-7 and MDA-MB-231 cells after treatment for 48 h [18]. Ginsenoside Rh2 induced apoptosis of breast cancer cells by inactivation of AKT [38]. Multiple triterpenoid saponins induced apoptosis in various other human cancer cells including gastric cancer [28], glioma [39] and choriocarcinoma [40] through inhibiting PI3K/AKT signaling pathway. DRβ-H exerted apoptotic activity on breast cancer cells through inhibition of PI3K/AKT pathway as the same as many kinds of ginsenosides.

DRβ-H up regulated the phosphorylation of ERK1/2 from 5 min to 6 h without effect on phosphorylation of JNK and P38. Furthermore, U0126 reversed DRβ-H-induced apoptosis through inhibiting phosphorylation of ERK1/2. The rapid phosphorylation of ERK1/2 after DRβ-H treatment suggested that activation of ERK1/2 played a key role in the early events of DRβ-H induced apoptosis. Four different Mitogen-activated protein kinases (MAPKs) have been described: ERK1/2, c-jun N-terminal kinases (JNK), P38 and ERK5. The best studied MAPK pathway is ERK1/2. This kinase cascade presents novel opportunities for the development of new cancer therapies [41]–[43]. This is the first time to report that triterpenoid saponins could exert apoptotic activity in breast cancer cells by activating phosphorylation of ERK. Although reports about apoptosis involving MAPKs by triterpenoid saponins in breast cancer are seldom, previous studies have showed that both oleane-type triterpenoid saponins and ginsenosides could induce apoptosis of cancer cells by activating phosphorylation of ERK. For instance, cytotoxic oleane-type triterpene saponins from *Glochidion eriocarpum* induced apoptosis by activation of ERK in colon cancer cells [36]. AD-1, a ginsenoside derivative, showed apoptotic activity on lung cancer cells via activation of ERK pathway [44]. Some widely used famous chemotherapeutics [45] or compounds from natural plants [46], [47] showed apoptotic activity via activating phosphorylation of ERK in cancer cells. Moreover, some triterpenoid saponins showed other kinds of anti-tumor activity via ERK phosphorylation in cancer cells. For example, enhanced ERK1/2 activity was involved in induction of macroautophagy by triterpenoid B-group soyasaponins in colon cancer cells [48]. Our results suggested that DRβ-H could induce apoptosis of breast cancer via activation of ERK pathway.

Overall, DRβ-H regulated PI3K/AKT and ERK signaling pathway, and activated pro-apoptotic members of Bcl-2 family, such as Bax and Bak, then changed permeabilization of the outer mitochondrial membrane, which released Apaf-1 and Cytochrome *C* from the inter membrane space into the cytosol, where they promoted caspase-9 and caspase-3 activation. Hence DRβ-H might inhibit growth and induce apoptosis of breast cancer by the way described above.

Triterpenoid saponins exhibit biologic activity to multiple cancers including breast cancer [19], esophageal cancer [29], colon cancer [49], lung cancer [50], liver cancer [51], ovarian cancer [52] and so on. Triterpenoid saponins are further classified into several types on the basis of the skeletal structures of the aglycones, e.g. dammarane type, oleanane type, lupane type, lanostane type, cucurbitane type and so on [53]. Ginsenosides, most of which are dammarane-type triterpenoids, have attracted much attention from worldwide and have been reported widely to showed anti-breast cancer activity in the last decades. Compared with the long history and widespread research on dammarane-type triterpenoids, studies of oleanane-type triterpenoid saponin are limited. This is the first report on the pro-apoptotic effects of DRβ-H, a novel oleanane-type triterpenoid saponin on breast cancer cells and its comprehensive apoptosis pathways.

Oriental medicinal herbs are rich sources of potential cancer chemopreventive and therapeutic agents, but require rigorous and systematic *in vitro* and *in vivo* pre-clinical evaluations as exemplified in the current work to transform traditional herbal practices into evidence-based medicine [54]. This study provides apoptotic activity and the mechanism for the anticancer properties of DRβ-H, a novel oleanane-type triterpenoid saponin and suggests that oleanane-type triterpenoid saponin might be a potential option for the drug development and treatment for breast cancer.

## Supporting Information

Figure S1 **DRβ-H inhibited the growth and induced apoptosis of MCF-10A cells and the effects were weaker than breast cancer cells.** (A) MTT assay of MCF-10A cells treated by DRβ-H. (B) Inhibition rate of breast cancer cells and MCF-10A cells treated by DRβ-H with various concentrations for 72 h. (C) The apoptosis rate of MCF-10A cells treated by DRβ-H measured by flow cytometry. (D) Early apoptosis cells of three independent experiments were shown in column statistics. (E) The apoptosis rate of breast cancer cells and MCF-10A cells treated by DRβ-H with various concentrations for 48 h. Data are mean ± SEM of three independent experiments. *p<0.05 vs. DRβ-H-untreated group. The # shows that DRβ-H had significantly more growth inhibitory effect on breast cancers cells than MCF-10A cells. The † shows that DRβ-H had significantly more pro-apoptotic effect on breast cancers cells than MCF-10A cells.
(TIF)

Figure S2 **DRβ-H induced mitochondria-mediated apoptosis of MCF-7 and MDA-MB-231 cells.** (A) Mitochondrial membrane potential ΔΨm (% of control) of cells treated with DRβ-H for 2 h, 6 h, 12 h of three independent experiments were shown in column statistics. (B, C) Expressions of mitochondrial Apaf-1 and Cytochrome *C* of cells treated with DRβ-H for 2 h, 6 h, 12 h or 24 h of three independent experiments were shown in column statistics. (D, E) Caspase 3, Caspase 9 positive rate of cells treated with DRβ-H with/without caspase inhibitors of three independent experiments were shown in column statistics. (F, G) Expressions of Bcl-2 family proteins of cells treated with 20, 30 µg/ml DRβ-H of three independent experiments were shown in column statistics. Data are mean±SEM of three independent experiments. *p<0.05, **p<0.01, ***p<0.001 vs. DRβ-H-untreated group. ^#^ *p*<0.05, ^##^ *p*<0.01,^ ###^ *p*<0.001 vs. DRβ-H-treated alone group.
(TIF)

Figure S3 **DRβ-H induced apoptosis of MCF-7 and MDA-MB-231 cells by inhibiting PI3K/AKT signaling pathway.** (A, B) Expressions of PI3K, p-PI3K, PDK1, p-PDK1, AKT and p-AKT of cells treated with DRβ-H for 5 min, 30 min, 2 h, 6 h, 48 h of three independent experiments were shown in column statistics. (C, D) Expressions of p-AKT of cells treated with DRβ-H and/or LY294002 for 48 h of three independent experiments were shown in column statistics. Data are mean±SEM of three independent experiments. *p<0.05, **p<0.01, ***p<0.001 vs. DRβ-H-untreated group. ^#^ *p*<0.05, ^##^ *p*<0.01,^ ###^ *p*<0.001 vs. DRβ-H-treated alone group.
(TIF)

Figure S4 **DRβ-H induced apoptosis of MCF-7 and MDA-MB-231 cells by activating ERK.** (A, B) Expressions of ERK, p-ERK, JNK, p-JNK, P38, p-P38 of cells treated with DRβ-H for 5 min, 30 min, 2 h, 6 h, 48 h of three independent experiments were shown in column statistics. (C, D) Expressions of p-ERK of cells treated with DRβ-H and/or U0126 for 30 min of three independent experiments were shown in column statistics. Data are mean±SEM of three independent experiments. *p<0.05, **p<0.01, ***p<0.001 vs. DRβ-H-untreated group. ^#^ *p*<0.05, ^##^ *p*<0.01,^ ###^ *p*<0.001 vs. DRβ-H-treated alone group.
(TIF)

Table S1 **Additional information for Western blotting analysis.**
(DOCX)
