# Supplementary figures and images for: The Apoptotic Effect of D Rhamnose β-Hederin, a Novel Oleanane-Type Triterpenoid Saponin on Breast Cancer Cells
Source: PLoS One. 2014 Mar 6;9(3):e90848. doi: 10.1371/journal.pone.0090848 (PMC3946269; doi:10.1371/journal.pone.0090848)

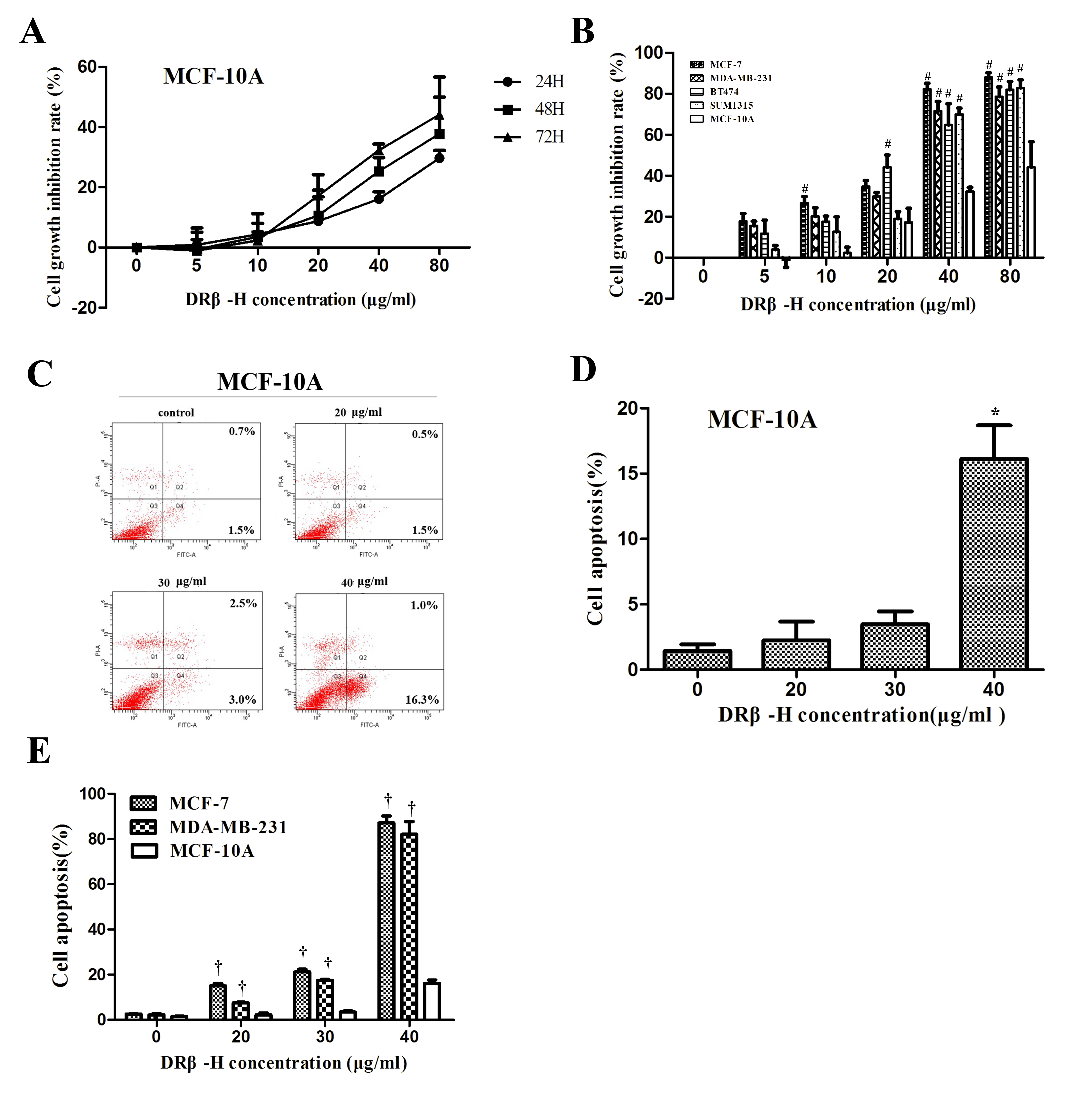

Supplement: Figure S1 — DRβ-H inhibited the growth and induced apoptosis of MCF-10A cells and the effects were weaker than breast cancer cells. (A) MTT assay of MCF-10A cells treated by DRβ-H. (B) Inhibition rate of breast cancer cells and MCF-10A cells treated by DRβ-H with various concentrations for 72 h. (C) The apoptosis rate of MCF-10A cells treated by DRβ-H measured by flow cytometry. (D) Early apoptosis cells of three independent experiments were shown in column statistics. (E) The apoptosis rate of breast cancer cells and MCF-10A cells treated by DRβ-H with various concentrations for 48 h. Data are mean ± SEM of three independent experiments. *p<0.05 vs. DRβ-H-untreated group. The # shows that DRβ-H had significantly more growth inhibitory effect on breast cancers cells than MCF-10A cells. The † shows that DRβ-H had significantly more pro-apoptotic effect on breast cancers cells than MCF-10A cells. (TIF) [file pone.0090848.s001.tif]

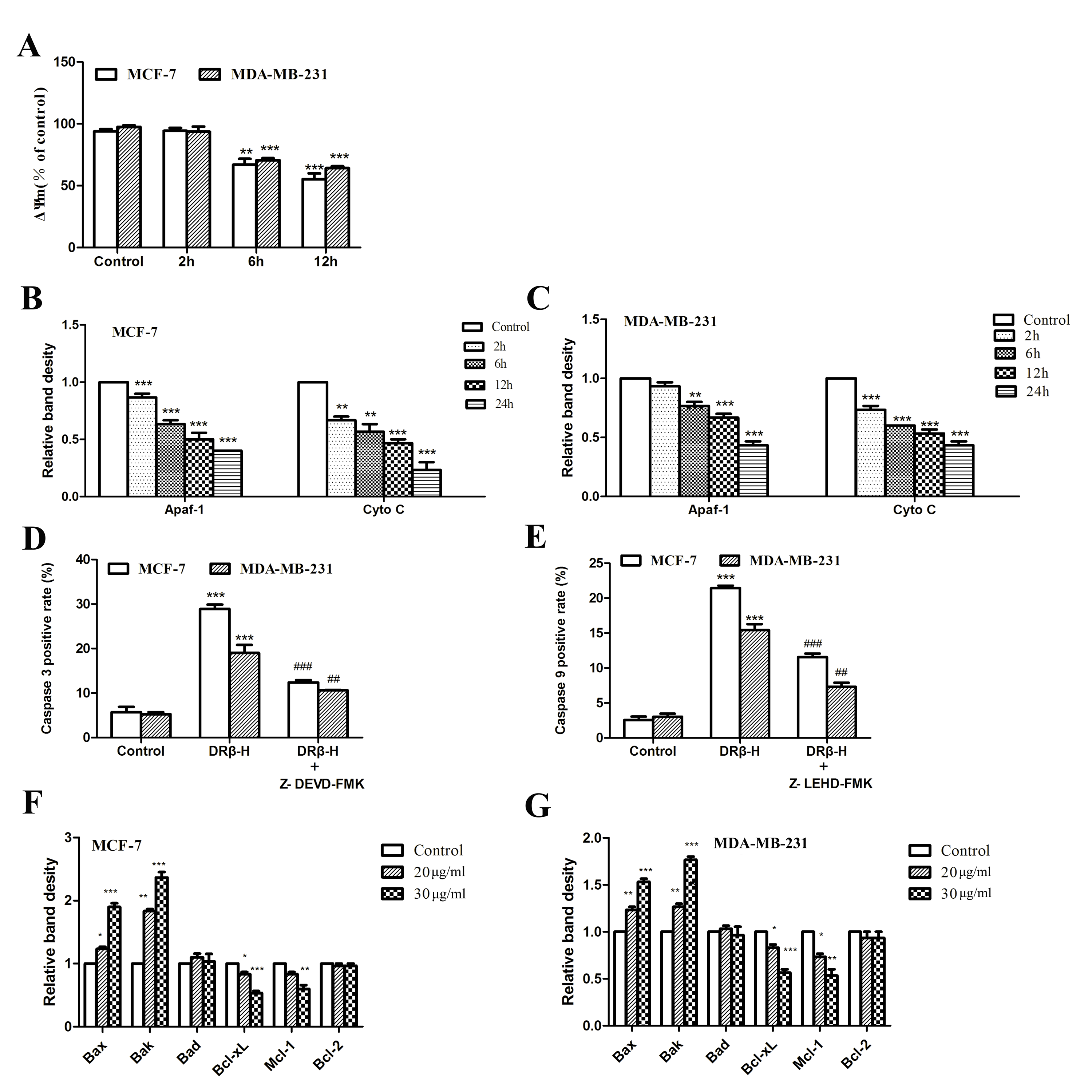

Supplement: Figure S2 — DRβ-H induced mitochondria-mediated apoptosis of MCF-7 and MDA-MB-231 cells. (A) Mitochondrial membrane potential ΔΨm (% of control) of cells treated with DRβ-H for 2 h, 6 h, 12 h of three independent experiments were shown in column statistics. (B, C) Expressions of mitochondrial Apaf-1 and Cytochrome C of cells treated with DRβ-H for 2 h, 6 h, 12 h or 24 h of three independent experiments were shown in column statistics. (D, E) Caspase 3, Caspase 9 positive rate of cells treated with DRβ-H with/without caspase inhibitors of three independent experiments were shown in column statistics. (F, G) Expressions of Bcl-2 family proteins of cells treated with 20, 30 µg/ml DRβ-H of three independent experiments were shown in column statistics. Data are mean±SEM of three independent experiments. *p<0.05, **p<0.01, ***p<0.001 vs. DRβ-H-untreated group. # p<0.05, ## p<0.01, ### p<0.001 vs. DRβ-H-treated alone group. (TIF) [file pone.0090848.s002.tif]

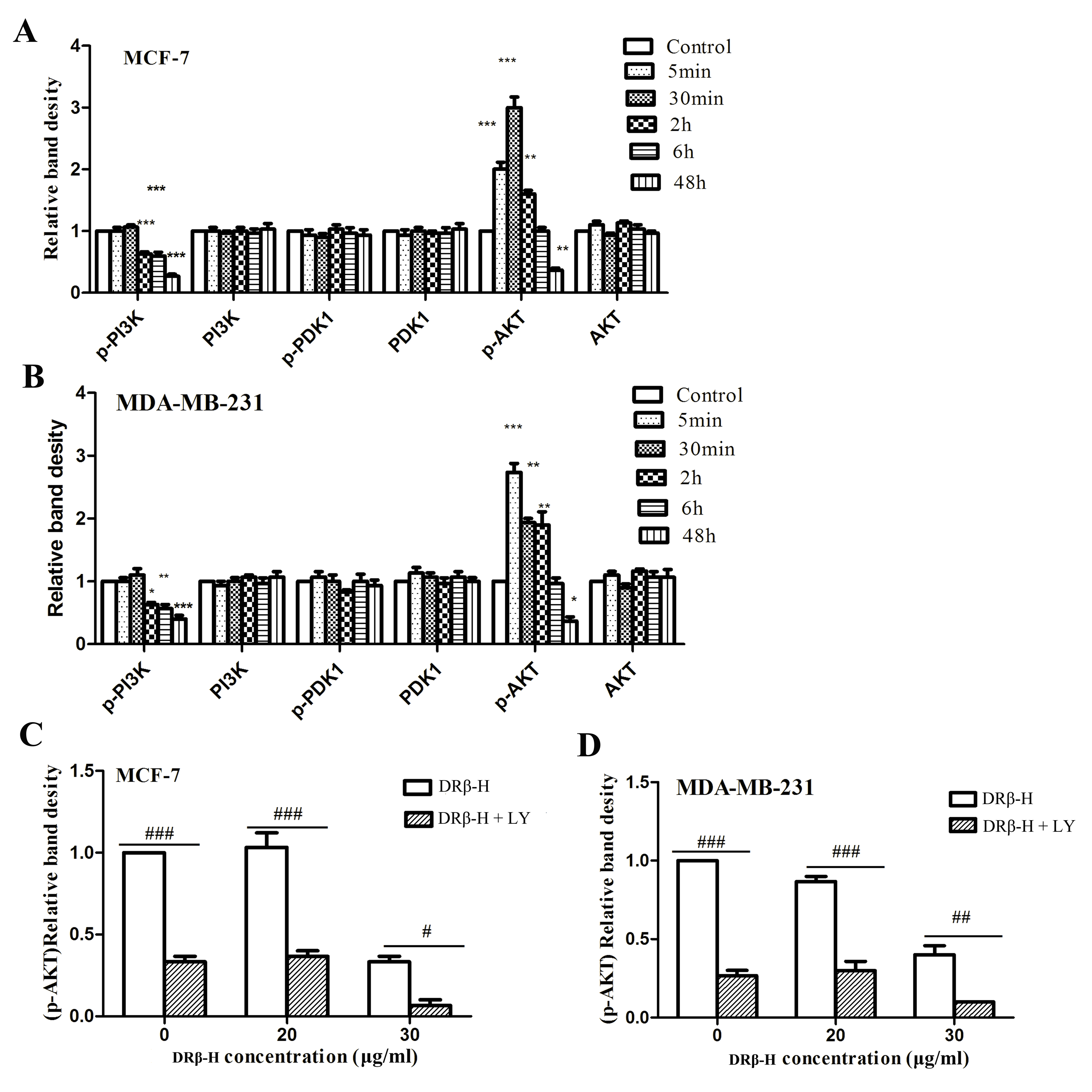

Supplement: Figure S3 — DRβ-H induced apoptosis of MCF-7 and MDA-MB-231 cells by inhibiting PI3K/AKT signaling pathway. (A, B) Expressions of PI3K, p-PI3K, PDK1, p-PDK1, AKT and p-AKT of cells treated with DRβ-H for 5 min, 30 min, 2 h, 6 h, 48 h of three independent experiments were shown in column statistics. (C, D) Expressions of p-AKT of cells treated with DRβ-H and/or LY294002 for 48 h of three independent experiments were shown in column statistics. Data are mean±SEM of three independent experiments. *p<0.05, **p<0.01, ***p<0.001 vs. DRβ-H-untreated group. # p<0.05, ## p<0.01, ### p<0.001 vs. DRβ-H-treated alone group. (TIF) [file pone.0090848.s003.tif]

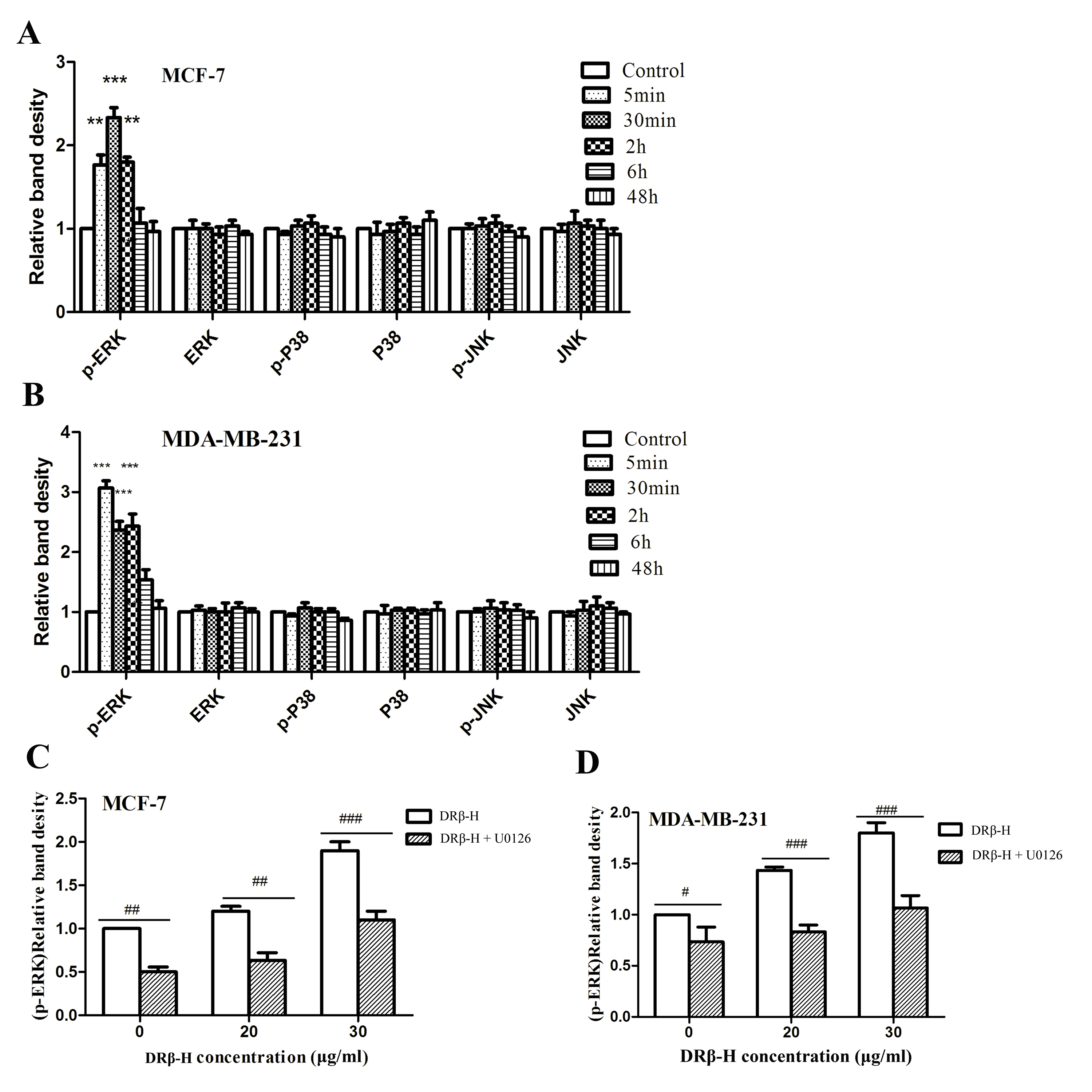

Supplement: Figure S4 — DRβ-H induced apoptosis of MCF-7 and MDA-MB-231 cells by activating ERK. (A, B) Expressions of ERK, p-ERK, JNK, p-JNK, P38, p-P38 of cells treated with DRβ-H for 5 min, 30 min, 2 h, 6 h, 48 h of three independent experiments were shown in column statistics. (C, D) Expressions of p-ERK of cells treated with DRβ-H and/or U0126 for 30 min of three independent experiments were shown in column statistics. Data are mean±SEM of three independent experiments. *p<0.05, **p<0.01, ***p<0.001 vs. DRβ-H-untreated group. # p<0.05, ## p<0.01, ### p<0.001 vs. DRβ-H-treated alone group. (TIF) [file pone.0090848.s004.tif]
