# Supplementary material for: The Apoptotic Effect of D Rhamnose β-Hederin, a Novel Oleanane-Type Triterpenoid Saponin on Breast Cancer Cells
Source: PLoS One. 2014 Mar 6;9(3):e90848. doi: 10.1371/journal.pone.0090848 (PMC3946269; doi:10.1371/journal.pone.0090848)
Supplement: Table S1 — Additional information for Western blotting analysis. (DOCX) [file pone.0090848.s005.docx]

**Table S1. Additional information for Western blotting analysis**

| Primary antibody | Blocking steps | Wash steps | Dilution  (primary antibody) | Incubation duration;  Temperature  (primary antibody) | Secondary antibodies | Dilution  (secondary antibody) | Incubation duration;  temperature  (secondary antibody) |
| --- | --- | --- | --- | --- | --- | --- | --- |
| PI3K, p-PI3K;  PDK1,p-PDK1;  AKT, p-AKT;  ERK1/2;p-ERK1/2;  JNK, p-JNK;  P38, p-P38  Bcl-2 family | 1 hour at room temperature | Three times for 5 minutes | 1:1000 | Over night at 4 ^o^C | Anti-rabbit | 1:1000 | 1 hour at room temperature |
| Apaf-1;  Cytochrome *C* | 1 hour at room temperature | Three times for 5 minutes | 1:1000 | Over night at 4 ^o^C | Anti-mouse | 1:1000 | 1 hour at room temperature |
| GAPDH | 1 hour at room temperature | Three times for 5 minutes | 1:1000 | Over night at 4 ^o^C | Anti-mouse | 1:1000 | 1 hour at room temperature |
